# Metabolic adaptation of the clam *Ruditapes philippinarum* during air exposure and the positive effects of sodium nitroprusside pretreatment

**DOI:** 10.3389/fphys.2023.1308777

**Published:** 2023-12-07

**Authors:** Zhilong Zheng, Zhongming Huo, Kaiyue Huang, Min Jiang, Xiwu Yan, Yang Liu, Yanjie Qin

**Affiliations:** Engineering and Technology Research Center of Shellfish Breeding in Liaoning Province, College of Fisheries and Life Science, Dalian Ocean University, Dalian, China

**Keywords:** *Ruditapes philippinarum*, air exposure, metabolomics, sodium nitroprusside, gill tissue structure

## Abstract

The Manila clam (*Ruditapes philippinarum*), as one of the shellfish living in the intertidal zone, is known for its strong ability to withstand air exposure. Sodium nitroprusside (SNP), a donor of nitric oxide (NO), has been shown to be useful for antioxidant and immune regulation in aquatic animals. In this study, an untargeted metabolomics (LC–MS/MS) technique was employed for the first time in Manila clam to analyze the metabolic and histological impacts after air exposure and the positive effects of SNP pretreatment. During air exposure, a significant increase in taurine, L-glutamate, and several polyunsaturated fatty acids in clams was detected, which indicates that clams may experience inflammatory reactions, oxidative stress, and an increase in blood ammonia content. When clams were exposed to SNP for 6 h, arginine, spermine, L-glutamic acid, and glutathione content were all upregulated, indicating that the SNP exposure induced NO production and improved antioxidant capacity in clams. When the clams were exposed to air after SNP pretreatment, there were no significant differences in the levels of taurine, L-glutamate, or aliphatic acids between the experimental and control groups. Gill tissue was more severely damaged in clams directly exposed to air than in those that experienced air exposure after SNP pretreatment, especially in clams exposed to air for a long time (72 h). Both metabolomics and tissue section structure indicated that SNP pretreatment decreased the stress responses caused by air exposure in *R. philippinarum*. These findings provided fresh insights and a theoretical foundation for understanding the tolerance to air exposure and physiological functions of SNP (or NO) in *R. philippinarum*.

## 1 Introduction

The Manila clam (*Ruditapes philippinarum*) is an economically important marine bivalve species in aquaculture, which has a widespread natural distribution in coastal areas from Europe to Asia. As a mollusc living in the intertidal zone, the Manila clam must deal with hard dynamic environments and environmental changes caused by physical elements such as PH, salinity, temperature, osmotic pressure, and air exposure (Zhang et al., 2015). Among these physicochemical factors, air exposure is one of the most influential factors. Intertidal organisms exposed to air could cause physiological responses that might lead to deaths in prolonged water-deficient circumstances (Li et al., 2017). The ability to survive in open air is a critical factor in the harvesting and transporting of many shellfish (Gu et al., 2020). During air exposure, clam shells are usually closed to reduce water loss to a minimum and protect the soft tissues (Coleman, 1973; Ali and Nakamura, 1999). However, due to an acidosis caused by anaerobic end-product formation or continuous aerobic metabolism, the prevention of breathing by tightly closing valves increases mortality during air exposure (Byrne et al., 1988; Ali and Nakamura, 2000). It was reported that *Meretrix meretrix* began to die after more than 24 h of air exposure (Wei et al., 2017). Mussels (*Perna perna*) exposed to air for 6, 12, 24, and 48 h showed hypoxia-induced antioxidant and tissue-specific immune systems (Nogueira et al., 2017). Significant oxidative stress can be caused by air exposure, which can result in oxidative damage and antioxidation (Xu et al., 2018). Oxidative stress can produce an increase in reactive oxygen species (ROS), which can cause substantial cell damage (Jia et al., 2019). A series of antioxidant defense mechanisms, including antioxidant enzymes, are produced in the organism to preserve its balance (Halliwell, 1999). Many studies have focused on the influence of air exposure on the molecular, physiological, and biochemical responses in *Crassostrea gigas* (Kawabe and Yokoyama, 2010; Gong et al., 2019), *Haliotis diversicolor* (Zhang et al., 2019), and *Solen grandis* (Nie et al., 2018). Recently, the effects of aerial exposure on the clams have been investigated in many aspects, including gene expression variation, metabolite changes, and physiological responses (Nie et al., 2020; Sun et al., 2021).

Nitric oxide (NO) is a key signaling chemical in marine invertebrates, and its signaling pathway is extremely conserved among species (Palumbo, 2005). NO has been demonstrated to have a wide range of biological roles in marine invertebrates, including eating, defense, and immunological responses (Palumbo, 2005), and to slow down the respiration of gills in bivalve mollusks (Gonzáleze et al., 2019). NO is catalyzed by monoxide synthase (NOS) in the presence of oxygen (O_2_), NADPH, and a substrate (L-arginine) (Förstermann and Sessa, 2012). In eukaryotes, there are three forms of NOS: neuronal NOS (nNOS), endothelial NOS (nNOS), and inducible NOS (iNOS). Among them, iNOS is considered to be induced by the stimulation of environmental perturbations (Kraft and Harry, 2011). A small quantity of NO can be produced by structured NOS under normal circumstances in organisms, and it is a chain blocker that can inhibit lipid peroxidation and scavenge oxygen free radicals (Yu et al., 2021). NO has also been proved to inhibit the production of mitochondrial oxygen free radicals in arctic clams (*Arctica islandica*) following chronic hypoxia and hypoxia (Strahl and Abele, 2020). Xian et al. (2013) used flow cytometry to evaluate NO production in *Penaeus monodon*, proving that the SNP (NO donor) could dramatically boost NO production in shrimp hemocytes. It was indicated that the supplementation of exogenous SNP and the synthesis of NO in marine organisms should be an effective way to improve antioxidant or immune capacity.

Metabolomics is a high-throughput molecular method that can quickly identify and quantify small-molecule metabolites (metabolomes) and is frequently considered the closest discipline to phenomics (Guijas et al., 2018). In recent years, this technology has gradually been applied to the study of the environmental adaptability of bivalve mollusks. Different metabolites in response to acute hypoxia in clams have been investigated with the goal of revealing the adaptive mechanism of acute hypoxia tolerance at the metabolic level (Sun et al., 2021). Metabolic profiles revealed that high temperatures caused changes in fatty acid composition, energy metabolism, antioxidant metabolites, hydroxyl compounds, and amino acids in heat-hardened clams compared to non-hardened clams (Zhang et al., 2023). Targeted metabolomics technology revealed the adaptation mechanism to high temperature, low oxygen, and compound stress at the metabolic level in the hard clam (*Mercenaria mercenaria*) (Hu et al., 2022). To our knowledge, the metabolites in response to air exposure in clams are still very lacking. The underlying metabolite mechanisms of air exposure tolerance and exogenous substances to enhance the ability to resist air exposure remain largely unknown in this commercially important species.

In this research, we studied the effects of air exposure and SNP exposure on tissue damage in *R*. *philippinarum*. The metabolites of clams after air exposure with/without SNP pretreatment were, respectively, analyzed using untargeted metabolomics (LC–MS/MS). Based on the metabolic characteristics analysis of clams in open air and in the presence of SNP, we will explore the changes in metabolic adaptation mechanisms of clams during air exposure after SNP pretreatment. This research provides new insights into the adaptation of the Manila clam to air exposure, as well as revealing the antioxidant protection of nitric oxide (NO).

## 2 Materials and methods

### 2.1 Experimental animals

Manila clams (average weight: 11.3 ± 0.5 g; average shell length: 37.5 ± 1.5 mm) were collected from the coastal area of Lvshun, Dalian, China. These clams were temporarily cultured in farming tanks at 15°C ± 1°C for a week. During this period, the seawater was changed and chlorella was fed once a day.

### 2.2 Air exposure with/without SNP pretreatment and sample collection

Sodium nitroprusside (SNP, NO donor, Biyuntian Biotechnology Co., Ltd., China) was dissolved in ultrapure water to make a 200 mg/mL mother solution, which was then diluted with seawater according to the experimental concentration.

A total of 180 clams were randomly collected and cultured in six separate tanks. Among them, three were experimental groups, and these clams were cultured in seawater with an SNP concentration of 100 μm/L for 6 h (N6) and then exposed to air for 72 h (NE). The other three groups were kept in normal seawater for 6 h (C0) and then in open air for 72 h (CE), which were considered the control groups. The gills were collected from the clams treated with SNP/- for 6 h and air exposure for 24, 48, and 72 h. Three individuals were sampled from each group at each period and promptly preserved with 4% paraformaldehyde for tissue sectioning.

A total of 360 clams were divided into treatment and control groups, with six parallels in each group. The clams in the two groups were cultured in normal seawater (C0) and then treated with 100 μm/L SNP for 6 h (N6). Subsequently, both groups were exposed to air for 72 h (CE and NE). 200–400 μL hemolymph was collected from clams in each group (C0, N6, CE, and NE), quickly frozen in liquid nitrogen, and then, stored at −80°C for metabonomics analysis. A mixture of 100 μL of the sample and 400 μL of an 80% methanol solution was vortexed and stored in ice water for 5 min. The supernatant was extracted via centrifugation at 15,000 g for 20 min at 4°C and diluted with mass spectrometry-grade water until the methanol level was 53%. The obtained solution was transferred to a sterile, enzyme-free EP tube and centrifuged at 15,000 g for 20 min at 4°C. The supernatants were stored at −80°C and transferred for LC–MS/MS analysis (Want et al., 2013).

### 2.3 Tissue sectioning and metabolite analysis

Gills fixed in 4% paraformaldehyde for histological observations were embedded in paraffin, sectioned into 7-μm slices and stained with hematoxylin–eosin. The slices were observed and photographed using the BX21 microscope and DP73 CCD camera (Olympus), respectively.

The UHPLC–MS/MS analyses were performed using a Vanquish UHPLC system (Thermo Fisher Scientific, Germany) coupled with an Orbitrap Q Exactive^TM^ HF mass spectrometer (Thermo Fisher Scientific, Germany) at Novogene Co., Ltd. (Beijing, China). Samples were injected into a Hypersil GOLD column (100 mm × 2.1 mm, 1.9 μm) using a 17-min linear gradient at a flow rate of 0.2 mL/min. The eluents for the positive polarity mode were eluent A (0.1% FA in water) and eluent B (methanol). The eluents for the negative polarity mode were eluent A (5 mM ammonium acetate, pH 9.0) and eluent B (methanol). The solvent gradient was set as follows: 2% B, 1.5 min; 2%–85% B, 3 min; 85%–100% B, 10 min; 100%–2% B, 10.1 min; and 2% B, 12 min. The Q Exactive^TM^ HF mass spectrometer was operated in the positive/negative polarity mode with a spray voltage of 3.5 kV, capillary temperature of 320°C, sheath gas flow rate of 35 psi, auxiliary gas flow rate of 10 L/min, S-lens RF level of 60, and auxiliary gas heater temperature of 350°C.

The raw data files generated by UHPLC–MS/MS were processed using the Compound Discoverer 3.1 software (CD3.1, Thermo Fisher Scientific) to perform peak alignment, peak picking, and quantitation for each metabolite. The peaks were matched with mzCloud (https://www.mzcloud.org/), mzVault, and MassList databases to obtain accurate qualitative and relative quantitative findings. The metabolites were annotated using the KEGG database (https://www.genome.jp/kegg/pathway.html), HMDB database (https://hmdb.ca/metabolites), and LIPID MAPS database (http://www.lipidmaps.org/). The metabolomics data processing software metaX (Wen et al., 2017) was used to convert the data in the multivariate statistical analysis section, and principal component analysis (PCA) and partial least squares discriminant analysis (PLS-DA) were performed to provide an overview of metabolic data, general clustering, trend, and outlier visualization and then obtain the contribution (VIP value) of each metabolite. The univariate analysis is based on the *t*-test to calculate the statistical significance (*p*-value) of each metabolite between the two groups and the fold change (FC) of the metabolite between the two groups, referred to as the FC value. VIP >1 and *p*-value <0.05 were the default criteria for differential metabolite screening. The volcano map was created using the R package ggplot2. Metabolites of interest may be selected by comprehending the three parameters of the metabolite: VIP value, log_2_ (fold change), and −log_10_ (*p*-value). The clustering heatmap was created using the R package Pheatmap, and the metabolite quantification data were standardized using the z-score. The statistically significant correlation between differential metabolites was calculated using cor. mtest () in R language, and a *p*-value <0.05 was considered statistically significant. The functions of these metabolites and metabolic pathways were studied using the KEGG database. The metabolic pathway enrichment of differential metabolites was performed. When the ratio was satisfied by x/n > y/N, the metabolic pathway was considered as enrichment, and when the *p*-value of the metabolic pathway was <0.05, the metabolic pathway was considered statistically significant enrichment.

## 3 Result

### 3.1 Damages to gill tissue during air exposure with or without SNP pretreatment

**FIGURE 1:**
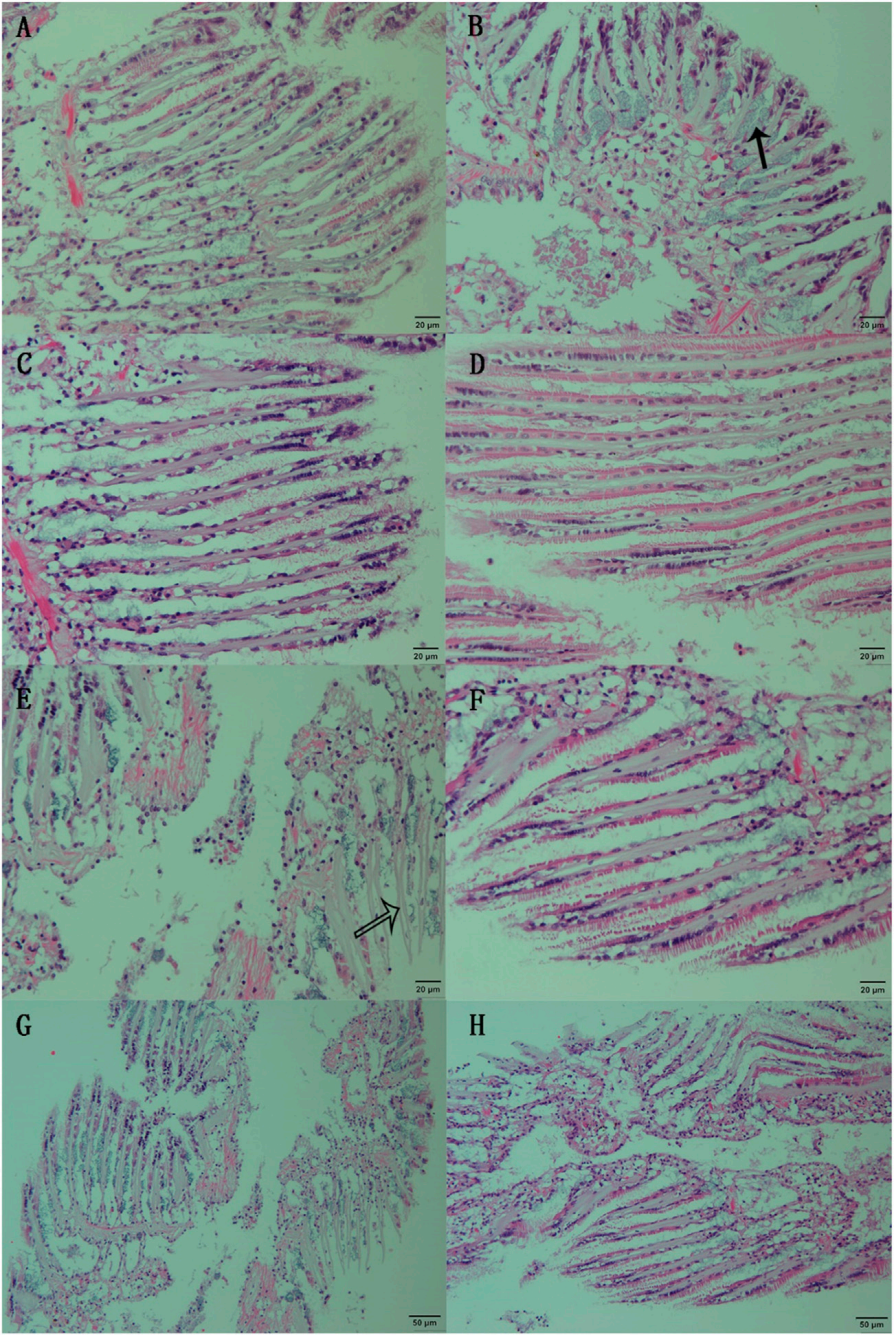
Histological observation of the gill of clam *Ruditapes philippinarum* undergoing air exposure and SNP pretreatment. **(A)** Gill in control (C0); **(B)** gill after 6 h of SNP treatment (N6); **(C)** gill after 24 h of air exposure (CE); **(D)** gill after 24 h of air exposure with SNP pretreatment (NE); **(E, G)** gill after 72 h of air exposure; and **(F, H)** gill after 72 h of air exposure with SNP pretreatment. Black arrow: clusters of mucous glands at the base of the gill filaments; white arrow: basal lamina after epithelial cell detachment.

Gill filaments in normal clams exhibited a dense arrangement, interconnected at their free ends by densely concentrated cilia. The gill filaments’ surface consisted of epithelial cells interspersed with goblets (Figure 1A). Following 6 h of SNP pretreatment, the fundamental tissue structure of the gill filaments remained unaltered. However, distinctive clusters of mucous glands appeared obviously at the base of the gill filaments (Figure 1B, marked by the black arrow). When the clams were exposed to air for 24 h, thinner gill filaments were observed, resulting in an increased inter-filament spacing and a notable proliferation of goblets within the epithelial cell layer (Figure 1C). After 72 h of air exposure, the gill tissue structure showed obvious damage (Figure 1G), with some epithelial cell detachment at the margin of gill filaments, thereby exposing the basal lamina (Figure 1E, indicated by the white arrow). When the clams were exposed to air for 24 h after a 6-h SNP pretreatment, the gill tissue structure remained intact and regular, as similar as those in control (Figure 1D). Even after 72 h of air exposure after SNP pretreatment, thinner gill filaments occurred and there was partial opening of inter-filament connections, resulting in increased inter-filament spacing. Nevertheless, the overall structural integrity remained, with minimal instances of epithelial cell detachment (Figures 1F, H).

### 3.2 Untargeted metabolome profiling

**FIGURE 2:**
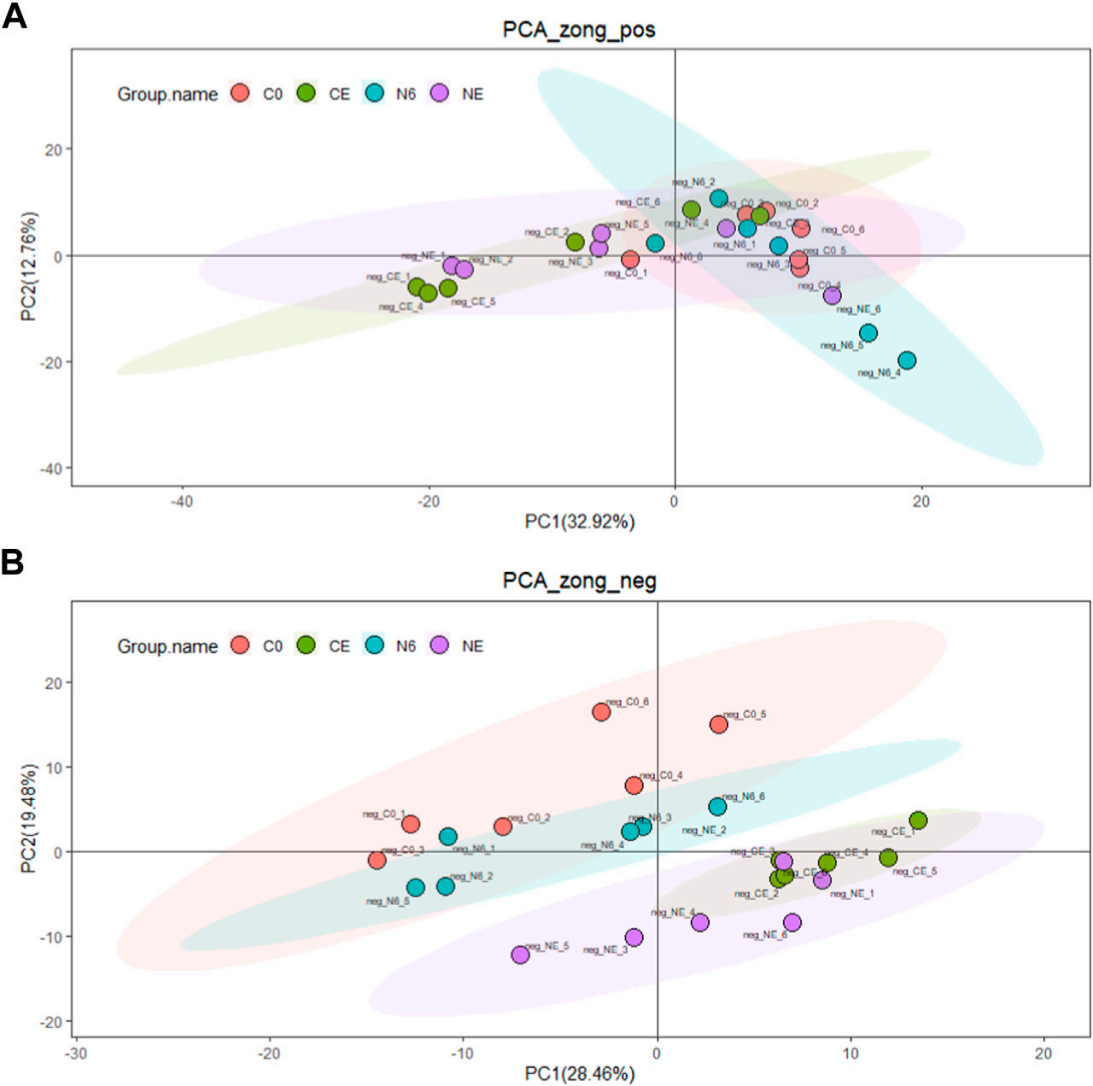
Principal component analysis of the clam *Ruditapes philippinarum* during air exposure with or without SNP pretreatment. **(A)** Positive ion (POS) and **(B)** negative ion (NEG). The red dots indicate the control group (C0), the green dots represent the air-exposed group (CE), the blue dots represent SNP exposure for 6 h (N6), and the purple dots represent the air-exposed clam after SNP pretreatment (NE).

Untargeted metabolomics analysis was used in this research to explore potential adaptation mechanisms at the metabolomics level. In all, 436 metabolites were found in the positive ion mode and 245 in the negative ion mode. PCA analysis was used to examine the changes in metabolites in each group (C0, N6, NE, and CE) (Figure 2). The PCA score chart displayed no significant outliers, showing that the four groups of metabolite aggregation are good. Furthermore, the overlap between the comparison groups is tiny, especially in the negative ion mode, demonstrating that the model has metabolite properties.

### 3.3 Analysis of differential metabolites

**FIGURE 3:**
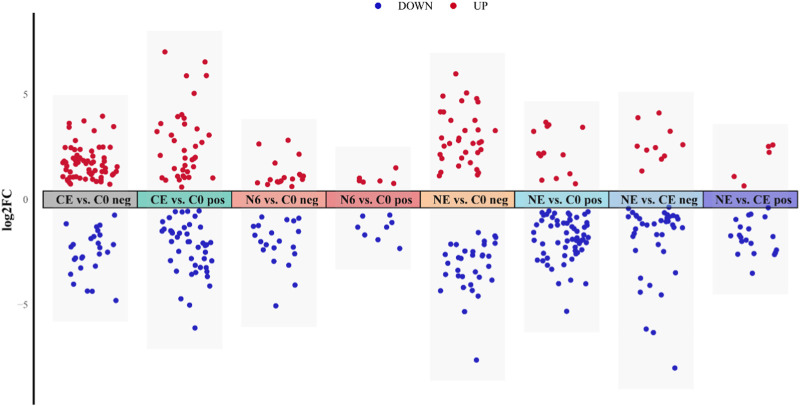
Metabolite visualization analysis of clam *Ruditapes philippinarum* exposed to SNP and air. Red dots represent upregulated metabolites, and blue dots represent downregulated metabolites.

In this research, VIP ≥1, FC > 1.2 or FC < 0.833, and *p*-value <0.05 were used as screening thresholds to analyze metabolite differences in the positive and negative ion modes (Figure 3). The analysis of the N6 vs. C0 comparison group showed that six significantly different metabolites (SDMs) were upregulated and eight SDMs were downregulated in the positive ion mode; 18 SDMs were upregulated and 20 SDMs were downregulated in the negative ion mode. In the NE vs. C0 comparison group, 13 SDMs were upregulated and 60 SDMs were downregulated in the positive ion mode; 34 SDMs were upregulated and 33 SDMs were downregulated in the negative ion mode. There were 37 upregulated and 44 downregulated SDMs in the positive ion mode and 67 upregulated and 26 downregulated SDMs in the CE vs. C0 comparison group. Results from the NE vs. CE comparison group showed that five upregulated and 20 downregulated SDMs were in the positive ion mode; 10 and 39 were upregulated and downregulated, respectively, in the negative ion mode.

**FIGURE 4:**
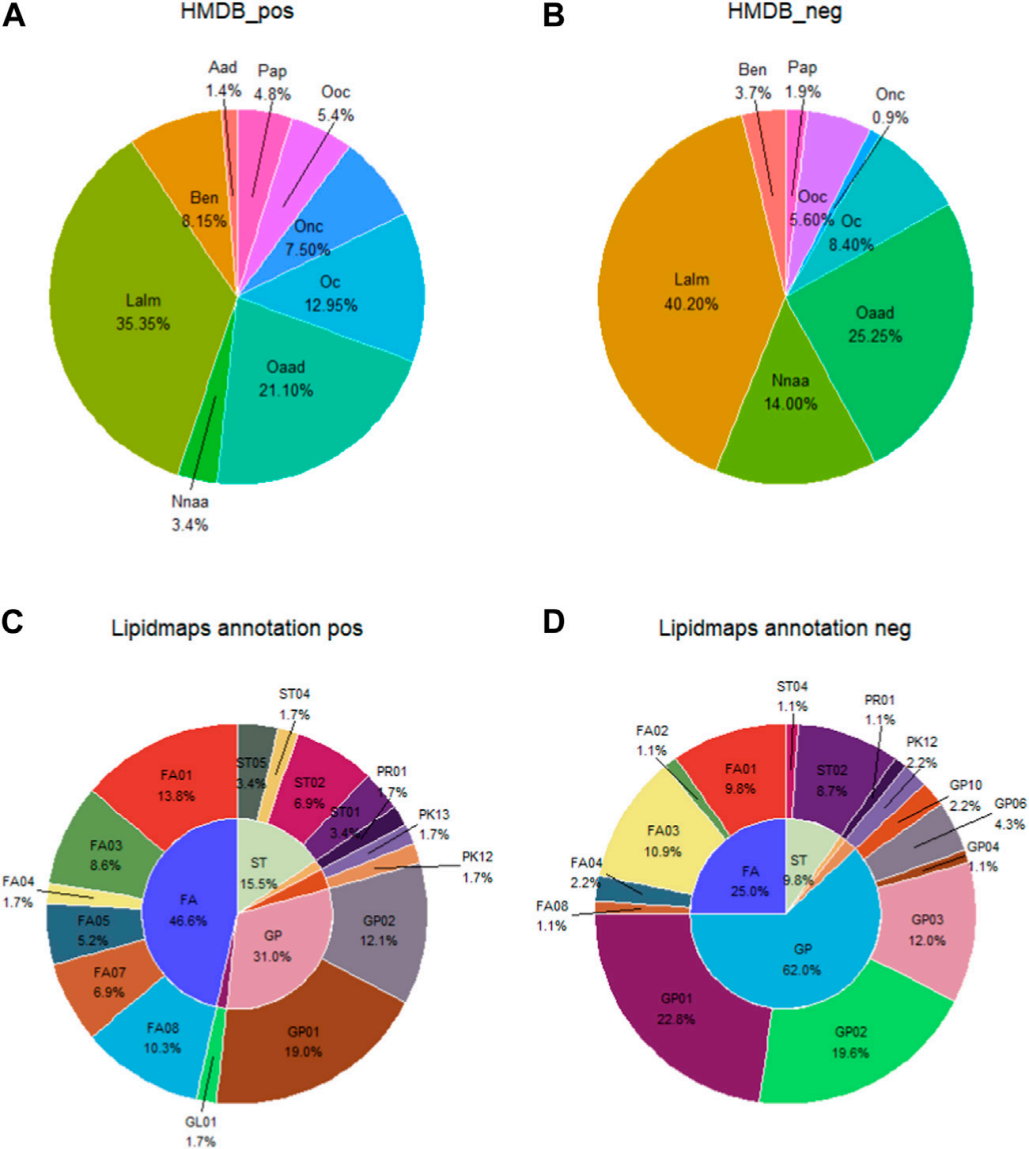
Account of metabolites annotated by the secondary classification (SuperClass) in HMDB **(A, B)** and the main level of classification (Main_Class) in LIPID MAPS **(C, D)**. Aad, alkaloids and derivatives; Ben, benzenoids; Lalm, lipids and lipid-like molecules; Nnaa, nucleosides nucleotides and analogs; Oaad, organic acids and derivatives; Oc, organoheterocyclic compounds; Onc, organic nitrogen compounds; Ooc, rganic oxygen compounds; Pap, phenylpropanoids and polyketides; FA, fatty acyls; ST, sterol lipids; GP, glycerophospholipids; FA01, fatty acids and conjugates; FA02, octadecanoids; FA03, eicosanoids; FA04, docosanoids; FA05, fatty alcohols; FA07, fatty esters; FA08, fatty amides; GL01, monoradylglycerols; GP01, glycerophosphocholines; GP02, glycerophosphoethanolamines; GP03, glycerophosphoserines; GP04, glycerophosphoglycerols; GP06, glycerophosphoinositols; GP10, glycerophosphates; PK12, flavonoids; PK13, aromatic polyketides; PR01, isoprenoids; ST01, sterols; ST02, steroids; ST04, bile acids and derivatives; and ST05, steroid conjugates.

In the HDMB database secondary classification (SuperClass), the metabolites in the positive and negative ion patterns could be categorized into nine groups: alkaloids and derivatives; benzenoids; lipids and lipid-like molecules; nucleosides/nucleotides and analogs; organic acids and derivatives; organoheterocyclic compounds; organic nitrogen compounds; organic oxygen compounds; and phenylpropanoids and polyketides. The proportion of each category is shown in Figures 4A, B. In the positive ion mode, the top three metabolite classifications of HDMB classification annotation findings were lipids and lipid-like molecules, organic acids and derivatives, and organoheterocyclic compounds. In the negative ion mode, the top three were lipids and lipid-like molecules, organic acids and derivatives, and nucleosides/nucleotides and analogs.

In the Lipid Metabolites and Pathways Strategy (LIPID MAPS) database, the metabolites identified in the positive and negative ion modes could be classified into six categories: 1) fatty acids (FAs); 2) glycerolipids (GLs); 3) glycerophospholipids (GPs); 4) polyketides (PKs); 5) prenol lipids (PRs); and 6) sterol lipids (STs). The percentages of each category are shown in Figures 4A, B. Fatty acids and conjugates [FA01], glycerophosphocholines [GP01], and glycerophosphoethanolamines [GP02] are the top three metabolite species in the positive ion mode. Glycerophosphocholines [GP01], glycerophosphoethanolamines [GP02], and glycerophosphosrines [GP03] are the top three metabolites in the negative ion mode (Figures 4C, D).

**FIGURE 5:**
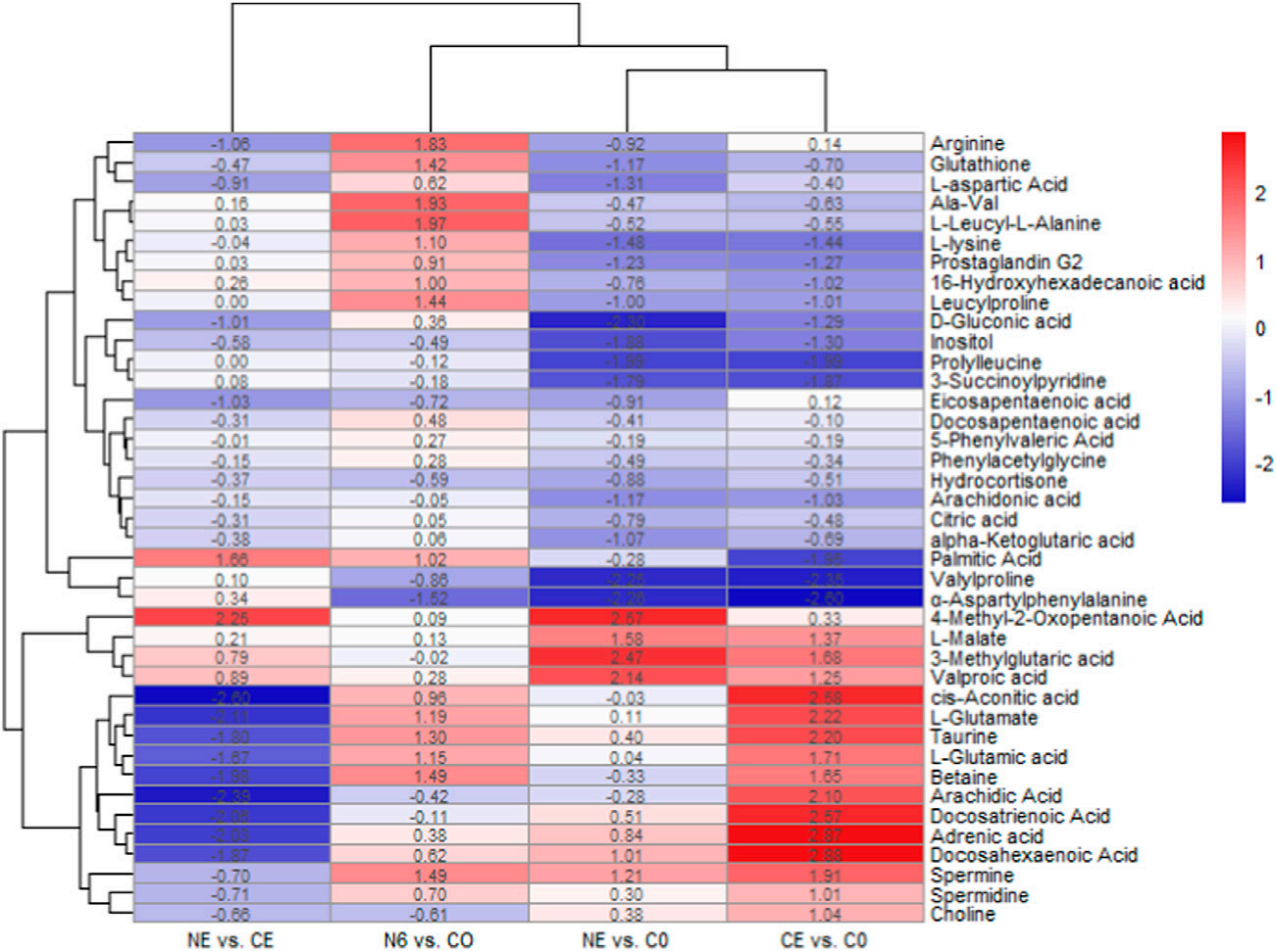
Clustering heatmap of metabolite expression after SNP pretreatment and air exposure in *Ruditapes philippinarum*. Metabolites in red are upregulated, and those in blue are downregulated.

The cluster heatmap indicated significant differences in metabolites between the groups, as shown in Figure 5. In the comparison between CE and C0 groups, ɑ-aspartylphenylalanine,valylproline, prolylleucine, palmitic acid, L-lysine, inositol, D-gluconic acid, glutathione, alpha-ketoglutaric acid, Ala-Val, citric acid, and L-aspartic acid were considerably downregulated, whereas cis-aconitic acid, taurine, spermine, L-glutamic acid, betaine, choline, and arginine were significantly upregulated. After 6 h of SNP exposure, arginine, spermine, and L-glutamic acid were all upregulated in the N6 group, indicating that the SNP exposure induced the production of NO in the body. It was also shown that the glutathione content was considerably higher in the N6 group. In the comparison between the NE and C0 groups, valylproline, ɑ-aspartylphenylalanine, D-gluconic acid, prolylleucine, and glutathione were all downregulated. However, taurine showed no significant changes. It was found that there were more upregulated metabolites in the CE vs. C0 group compared to the NE vs. C0 group.

**FIGURE 6:**
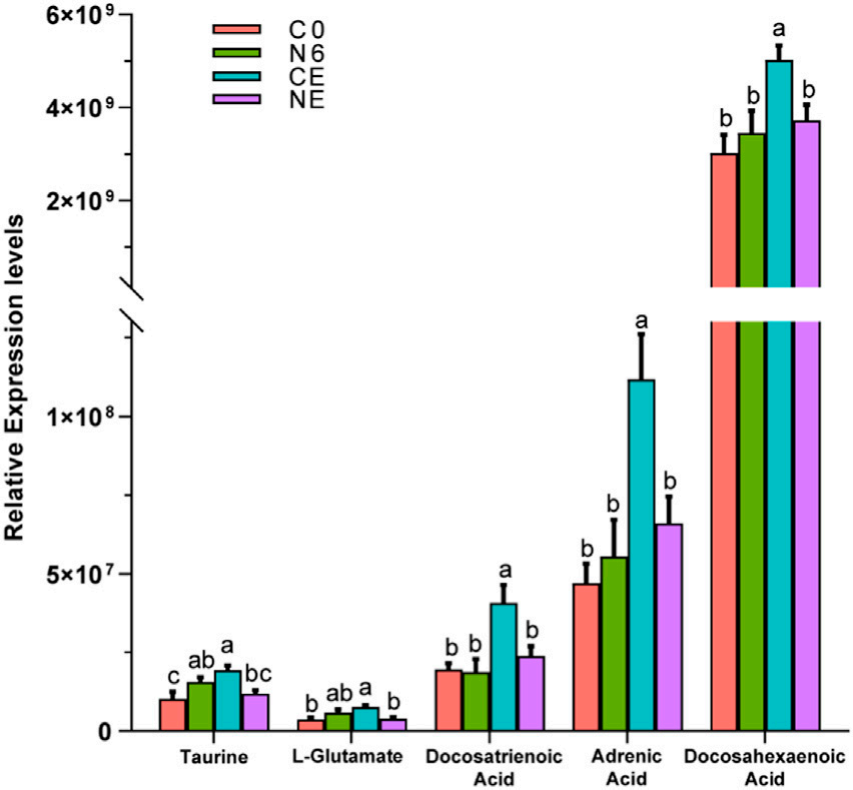
Comparative analysis of some metabolite expression levels in *Ruditapes philippinarum*. Different lowercase letters indicate significant differences in the levels of the same metabolite among distinct experimental groups.

The relative levels of some metabolites, such as taurine, L-glutamate, and some aliphatic acids, are depicted in Figure 6. These metabolites showed the most significant changes in the air-exposed clam, all of which were higher than those in the control group (*p* < 0.05). However, there were no significant differences in these metabolites between the NE and C0 groups (*p* > 0.05).

### 3.4 Analysis of KEGG pathway enrichment

**FIGURE 7:**
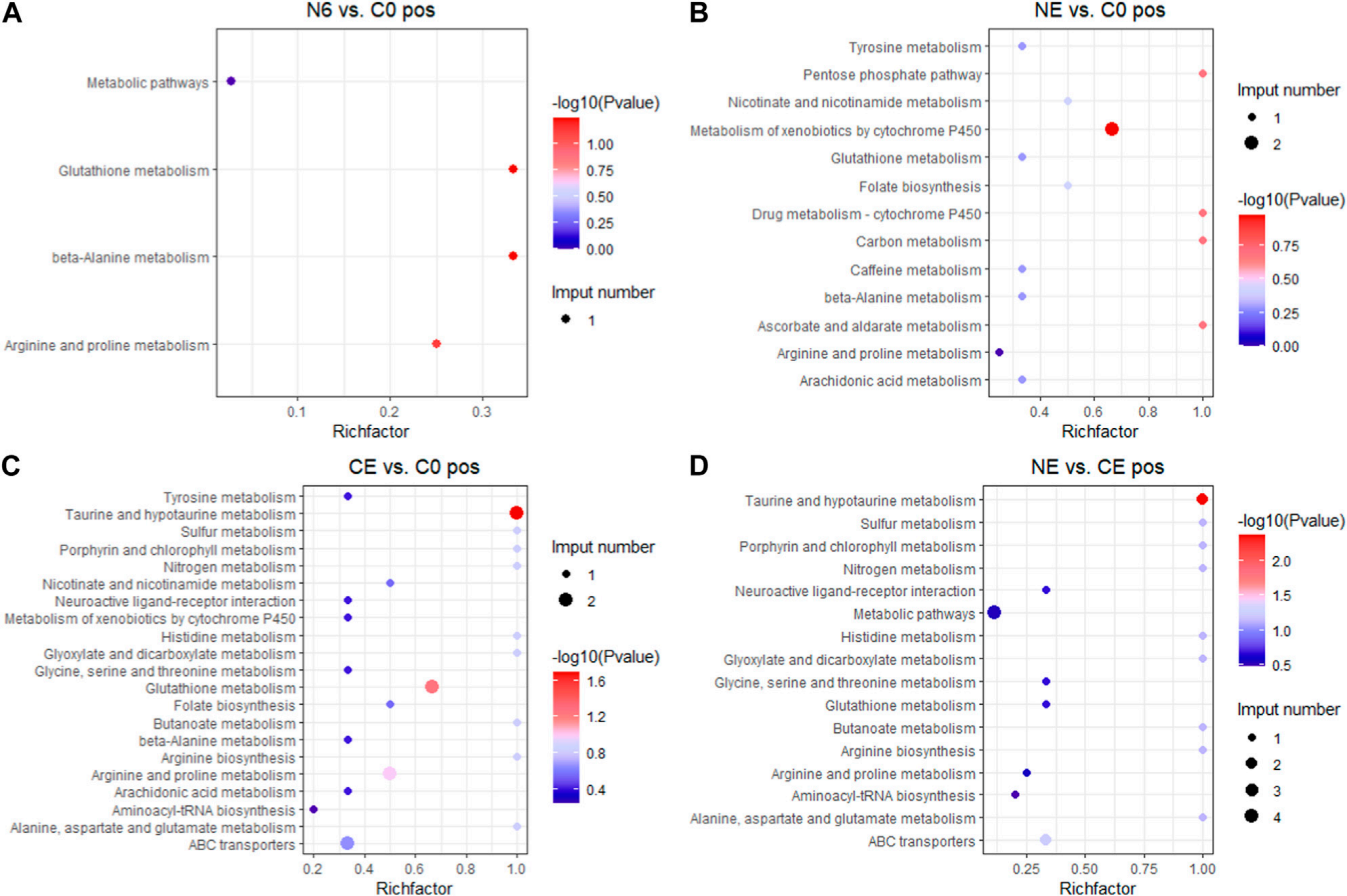
KEGG enrichment analysis in the positive ion mode across comparison groups. The larger the abscissa value, the greater the degree of enrichment of differential metabolites. The point’s color shows the *p*-value of the hypergeometric test. The lower the value is, the more reliable the test is and the more statistically significant it is. The size of the dots represents the number of different metabolites in the pathway. **(A)** N6 vs. C0; **(B)** NE vs. C0; **(C)** CE vs. C0; and **(D)** NE vs. CE.

**FIGURE 8:**
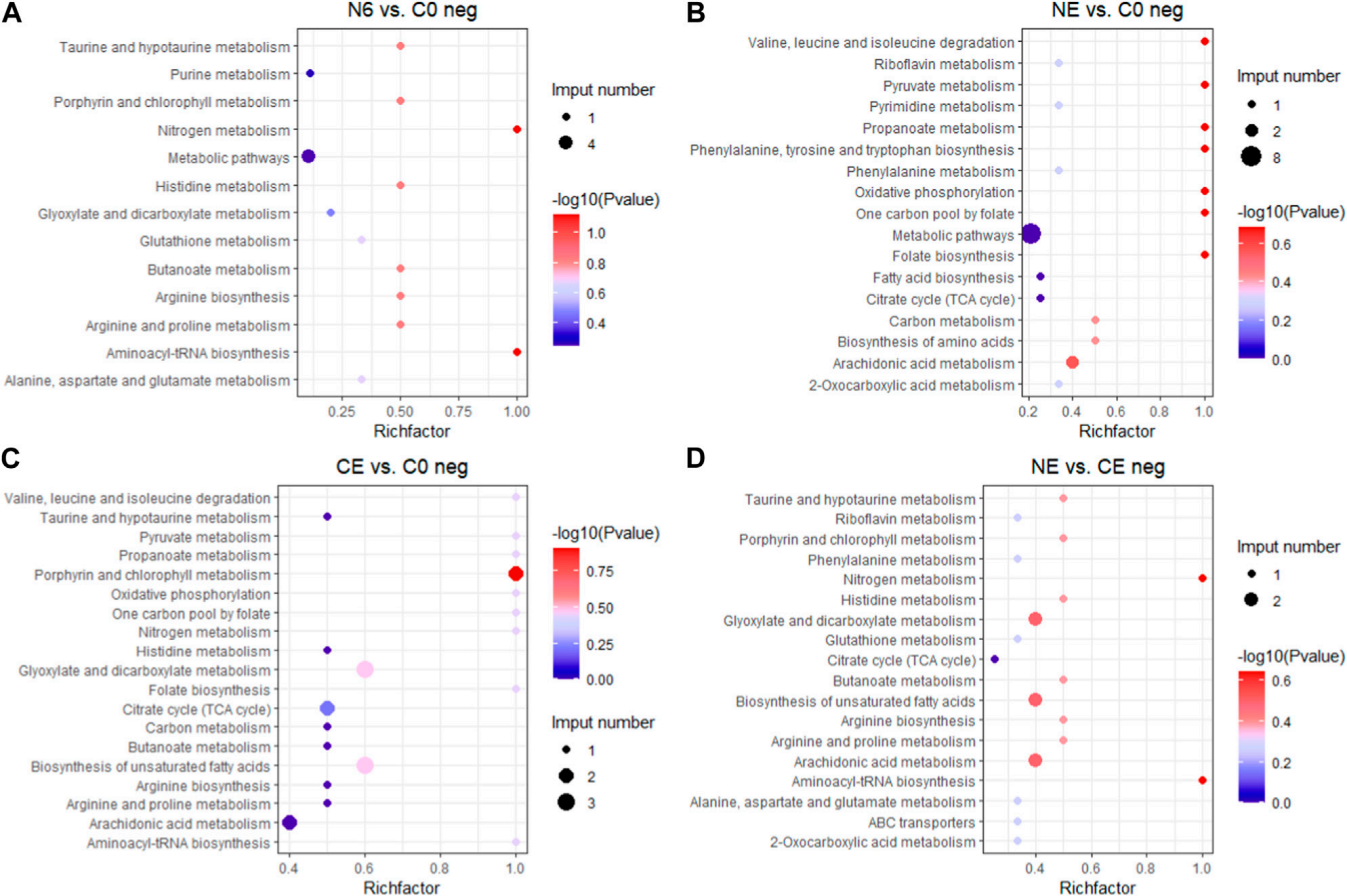
KEGG enrichment analysis in the negative ion mode across comparison groups. The larger the abscissa value, the greater the degree of enrichment of differential metabolites. The point’s color shows the *p*-value of the hypergeometric test. The lower the value is, the more reliable the test is and the more statistically significant it is. The size of the dots represents the number of different metabolites in the pathway. **(A)** N6 vs. C0; **(B)** NE vs. C0; **(C)** CE vs. C0; and **(D)** NE vs. CE.

To better understand the functions of different metabolites, SDMs in the four comparison groups were annotated into the KEGG pathway for enrichment analysis. The results showed that the SDMs in the N6 vs. C0 group were enriched in beta-alanine metabolism, arginine and proline metabolism, and glutathione metabolism in the positive ion mode (pos) (Figure 7A) and glutathione metabolism, arginine biosynthesis, and aminoacyl-tRNA biosynthesis enrichment in the negative ion mode (neg) (Figure 8A). In the positive ion mode (pos) of the NE vs. C0 group, SDMs were enriched in the biosynthesis of unsaturated fatty acids, glutathione metabolism, and the pentose phosphate pathway (Figure 7B), whereas the negative ion mode was enriched in oxidative phosphorylation and pyruvate metabolism (Figure 8B). The CE vs. C0 group was enriched in taurine and hypotaurine metabolism, glutathione metabolism, arginine and proline metabolism, and arginine biosynthesis in the positive ion mode (Figure 7C), and in the negative ion mode, biosynthesis of unsaturated fatty acids and oxidative phosphorylation were enriched (Figure 8C). The SDMs in the NE vs. CE group were enriched in taurine and hypotaurine metabolism, ABC transporters, and arginine biosynthesis in the positive ion mode (Figure 7D), and in the negative ion mode (neg), enrichment was observed in arginine biosynthesis, taurine and hypotaurine metabolism, and glutathione metabolism (Figure 8D).

## 4 Discussion

The LC–MS metabolomics technique was used for the first time in this research to determine the metabolic alterations of hemolymph after air exposure and SNP (NO donor) exposure in the Manila clam. The different metabolites in clams after air exposure and SNP treatment were mainly energy metabolism, osmoregulation, and the effects of oxidative stress. Air exposure was found to have an influence on important metabolic pathways such as amino acid metabolism, fatty acid metabolism, neurotransmitter transport, and osmoregulation (Liang et al., 2020). The cluster heatmap revealed that the amounts of various amino acids (prolylleucine, Ala-Val, leucylproline, L-aspartic acid, L-lysine, 5-phenylvaleric acid, citric acid, and alpha-ketoglutarate) dropped in clams exposed to air for 72 h. Amino acids have been shown to be a major source of energy metabolism in mollusks (Alfaro et al., 2021). A reduction in amino acids in bivalve mollusks suggests physiological changes in responses to several stresses. The contents of various amino acids decreased to different degrees under environmental stressors, such as high temperatures (Zhang et al., 2023), high temperatures and hypoxia (Hu et al., 2022), and air exposure (Nguyen et al., 2020). These results were consistent with our findings, which indicated that clams had substantial variations in metabolome profiles after air exposure. Long-term air exposure also causes oxygen deprivation in clams, which in turn affects several energy-related metabolites. The TCA cycle is an important pathway for the generation of energy in the animal body. TCA intermediate accumulation has been demonstrated to be caused by TCA cycle interruption in mollusks (Van Nguyen and Alfaro, 2019). When New Zealand surf clams (*Crassula aequilatera*) are exposed to air and high temperatures, they generate oxidative stress and an accumulation of TCA metabolic intermediates, with L-malate accumulating continuously (Alfaro et al., 2019). Similarly, the content of citric acid and alpha-ketoglutarate acid in clams exposed to air decreased continually in order to produce energy for the body, while the content of L-malate and cis-aconitic acid increased, which confirmed that the TCA cycle was interrupted in clams during air exposure.

It should be noted that the contents of docosapentaenoic acid, docosahexaenoic acid, arachidic acid, L-glutamic acid, and taurine were significantly increased in clams during air exposure. Among them, taurine is considered an antioxidant and an osmotic agent in cellular metabolism and steady state (Schaffer and Kim, 2018). Its antioxidation includes binding peroxidation reaction products (Ahmed, 2023), inhibiting mitochondrial superoxide production (Jong et al., 2012) and preventing reactive oxygen species (ROS) through the destruction of antioxidant enzymes. Taurine in clam hemolymph stayed at a high level during air exposure in this research, which indicated that increased taurine expression might be a protective mechanism against oxidative damage in clams during air exposure. Similarly, Haider et al. (2020) also found in their research that the high survival rates of marine bivalves during hypoxic stress were correlated with high levels of taurine. In addition, taurine is related to the osmoregulatory regulation in marine mollusk during environmental changes (Hosoi et al., 2007). The taurine-related metabolism pathway has been proved to have a non-negligible role in mollusk osmoregulation under hypoxic stress (Sun et al., 2021). During air exposure, we obviously detected a significant increase in taurine and other osmolytes, including non-essential amino acids, which indicated that clams faced alterations in osmoregulation. It was concluded that taurine acted as an antioxidant and osmotic agent in the Manila clam during air exposure. It was reported that the blood ammonia would combine with L-glutamate to form the amide (Pequin and Serfaty, 1966). Therefore, the increase in L-glutamate in clams in the CE group in this study might indicate a higher blood ammonia level during the air exposure. In addition, it was found in this study that some polyunsaturated fatty acids (PUFAs), such as docosatrienoic acid, adrenic acid, docosahexaenoic acid, and arachidic acid, significantly increased in air-exposed clams. There is substantial evidence that these fatty acids are capable of partly inhibiting many aspects of inflammation, including leukocyte chemotaxis, adhesion molecule expression, and leukocyte–endothelial adhesive interactions (Calder, 2017). The increase in PUFAs in the results of this study indicated that clams might experience inflammatory reactions during the air exposure process.

NO can directly alleviate oxidative stress at low concentrations by impacting the membrane components of NADPH oxidase and reducing the production of ROS (Wink et al., 2001; Clancy et al., 1992). González et al. (2019) discovered that mussels in hypoxic circumstances produced NO to achieve vasodilation. Moreover, as oxygen contents decreased after shell closure, nitric oxide produced from the hemolymph induced physiological damage to the gills and lowered the metabolic rate of other tissues. This reveals a basal function of NO in improving perfusion of hypoxic invertebrate tissues, which could be a key mechanism of tolerance toward environmental O_2_ variations. NO was proved to protect cells by reducing intracellular CytO_x_ and O_2_ uptake during long-term hypoxia, and thus, arctic clams will not undergo ROS bursts or antioxidant upregulation during hypoxia and recovery (Strahl and Abele, 2020; Strahl et al., 2011). The glutathione content rose substantially after Manila clams were exposed to SNP for 6 h. Kuo et al. (1996) discovered that when faced with nitric oxide-mediated oxidative stress, GSH production was induced in order to maintain appropriate antioxidant levels. When the formation rate of the superoxide anion (O^−2^) is constant, glutathione oxidation increases as NO increases. When the formation rate of NO is greater than that of these free radicals, GSH oxidation weakens and glutathione concentrations increase significantly (Wink et al., 2001). Both might account for the large increase in glutathione levels after SNP exposure. The results of this experimental research also revealed that the related antioxidant substances (such as taurine and glutathione) were significantly upregulated in SNP exposure experiments. These results indicated that NO can not only directly exert antioxidant effects but also induce the generation of antioxidant substances (such as taurine and glutathione), jointly achieving antioxidant function. When the clams were exposed to air after 6 h of SNP pretreatment (NE group), compared to the control group (C0), the taurine value was not significantly changed, while glutathione was at a reduced level. It was speculated that after SNP treatment, NO in the clam body can continue to exert antioxidant effects for some time, and the large amount of taurine and glutathione induced by NO can also resist the oxidative reaction during air exposure. Therefore, during the air exposure after SNP pretreatment (NE), most metabolites that significantly increased in the directly air-exposed group (CE), such as taurine, L-glutamate, and several PUFAs, showed no significant differences from those in the control group (C0).

Gill is an important organ for respiration and filtration, especially for bivalves (Zhan et al., 2021). In the present study, the structures of the gill of clams were remarkably destroyed after 72 h of air exposure. Large amounts of oxygen free radicals (ROS) would be produced in the gill due to the insufficient supply of oxygen molecules (Kim and Chung, 2007; Jing et al., 2023). The rupture of gill cells in clams during air exposure may be caused by excessive ROS production under respiratory obstruction and hypoxia. However, compared to the direct air exposure group, gill tissue was only slightly destroyed in clams exposed to air for 72 h after SNP pretreatment. Combined with the results of metabolomics, it was indicated that the stress response caused by air exposure in clams would be decreased after SNP (NO donor) pretreatment.

## 5 Conclusion

This research is the first to use untargeted metabolomics (LC–MS/MS) to analyze the adaptive mechanism of Manila clams during air exposure and the positive effect of SNP (NO donor) pretreatment before air exposure. A significant increase in taurine, L-glutamate, and several PUFAs in the Manila clam was detected, which indicated that clams may experience inflammatory reactions, oxidative stress, and an increase in the blood ammonia content during air exposure. NO induced by SNP could induce the production of taurine and glutathione in the clam. When the clams were exposed to air after 6 h of SNP pretreatment, NO could not only directly exert antioxidant effects but also induce the generation of antioxidant substances (such as taurine and glutathione), jointly achieving antioxidant function. Compared with the serious damages in the gills of clam directly exposed to air, gill tissue was only slightly destroyed in clams exposed to air for 72 h after SNP pretreatment. Therefore, it was indicated that a series of physiological responses during air exposure in *Ruditapes philippinarum* could be alleviated through SNP pretreatment.

## Data Availability

The original contributions presented in the study are included in the article/supplementary material; further inquiries can be directed to the corresponding author.
